# Urosepsis 30-day mortality, morbidity, and their risk factors: SERPENS study, a prospective, observational multi-center study

**DOI:** 10.1007/s00345-024-04979-2

**Published:** 2024-05-10

**Authors:** Zafer Tandogdu, Bela Koves, Slobodan Ristovski, Mustafa Bahadir Can Balci, Kristin Rennesund, Stavros Gravas, DjordJe Nale, José Medina-Polo, Mária Kopilec Garabášová, Elisabetta Costantini, Jorge Cano-Valasco, Maja Sofronievska Glavinova, Franck Bruyere, Tamara Perepanova, Ekaterina Kulchavenya, Mete Cek, Florian Wagenlehner, Truls Erik Bjerklund Johansen, SERPENS Investigators

**Affiliations:** 1 University College London Hospitals, London, UK; 2 Division of Surgery and Interventional Science, University College London, Charles Bell Housr, London, UK; 3 South Pest Teaching Hospital, Budapest, Hungary; 4 University Clinic for Surgical Diseases “St. Naum Ohridski” Skopje, Skopje, Republic of North Macedonia; 5 Gaziosmanpasa Taksim Teaching Hospital, Istanbul, Turkey; 6 Urology Department, Oslo University Hospital, Oslo, Norway; 7 University Hospital of Larissa, Larissa, Greece; 8 Clinic of Urology, University Clinical Center of Serbia, Faculty of Medicine, Belgrade, Serbia; 9 Hospital Universitario, 12 de Octubre imas12, Madrid, Spain; 10 Teaching Hospital Trenčín, Trenčín, Slovakia; 11 Ospedale Santa Maria della Misericordia di Perugia, Perigia, Italy; 12 Hospital General Universitario Gregorio Marañón, Madrid, Spain; 13 Surgical Clinic St. Naum Ohridski, Skopje, Republic of North Macedonia; 14 Urology, CHRU Bretonneau, Tours, France; 15 S.R. Institute of Urology, Moscow, Russian Federation; 16 Novosibirsk Research Institute of Tuberculosis, Novosibirsk, Russian Federation; 17 Department of Urology, Trakya University Medical School, Edirne, Turkey; 18 Justus Liebig University, Giessen, Germany; 19 University of Oslo, Oslo, Norway; 20 Institute of Clinical Medicine, University of Aarhus, Aarhus, Denmark

**Keywords:** Severe UTI, Urosepsis, Antibiotic resistance

## Abstract

**Purpose:**

To provide a descriptive report of mortality and morbidity in the first 30 days of diagnosis of urosepsis. Secondary aim is to identify risk factors of unfavourable outcomes.

**Methods:**

Prospective observational multicentre cohort study conducted from September 2014 to November 2018 in European hospitals. Adult patients (≥ 18 years) diagnosed with acute urosepsis according to Sepsis-2 criteria with confirmed microbiological infection were included. Outcomes were classified in one of four health states: death, multiple organ failure, single organ failure, and recovery at day 30 from onset of urosepsis. Descriptive statistics and ordinal logistic regression analysis was performed.

**Results:**

Three hundred and fifty four patients were recruited, and 30-day mortality rate was 2.8%, rising to 4.6% for severe sepsis. All patients who died had a SOFA score of ≥ 2 at diagnosis. Upon initial diagnosis, 79% (*n* = 281) of patients presented with OF. Within 30 days, an additional 5% developed OF, resulting in a total of 84% affected. Charlson score (OR 1.14 CI 1.01–1.28), patients with respiratory failure at baseline (OR 2.35, CI 1.32–4.21), ICU admission within the past 12 months (OR 2.05, CI 1.00–4.19), obstruction causative of urosepsis (OR 1.76, CI 1.02–3.05), urosepsis with multi-drug-resistant(MDR) pathogens (OR 2.01, CI 1.15–3.53), and SOFA baseline score ≥ 2 (OR 2.74, CI 1.49–5.07) are significantly associated with day 30 outcomes (OF and death).

**Conclusions:**

Impact of comorbidities and MDR pathogens on outcomes highlights the existence of a distinct group of patients who are prone to mortality and morbidity. These findings underscore the need for the development of pragmatic classifications to better assess the severity of UTIs and guide management strategies.

*Study registration*: Clinicaltrials.gov registration number NCT02380170.

**Supplementary Information:**

The online version contains supplementary material available at 10.1007/s00345-024-04979-2.

## Introduction

Urosepsis, a commonly occurring form of sepsis resulting from urinary tract infections (UTIs), poses threat to life and can lead to long-term morbidity. UTIs are one of the most common sources of sepsis, with estimates ranging from 20 to 40% of all sepsis cases [1, 2]. Despite urosepsis having relatively low mortality rates, it remains an area that requires further research [2]. Our understanding of predictors of mortality and morbidity in urosepsis is limited, but identifying patient factors associated with urosepsis at the time of diagnosis holds promise for improving patient care and outcomes.

Urosepsis outcomes vary among populations and are influenced by factors like severity, patient fitness, frailty, and age [1,3,4]. Recent studies suggest that clinical findings can improve prognostic tools and aid decision-making [5]. While common risk factors for urosepsis include indwelling catheters, obstructive uropathy, tissue necrosis, abscess, urinary tract interventions, and urological functional impairments, their precise impact on outcomes remains uncertain [6].

Guidelines now favour a risk-based approach over using the Systemic Inflammatory Response Syndrome (SIRS) criteria for sepsis diagnosis and prognosis [4–6]. The low specificity of the SIRS criteria have contributed to potential inclusion of non-infectious conditions and overdiagnosis of sepsis [5]. Despite poor specificity, SIRS remains widely used due to its simplicity. However, performance programs for sepsis have improved early detection and outcomes [7]. Furthermore, the rise in antimicrobial resistance (AMR) and multi-drug resistant (MDR) pathogens compromises effectiveness of antibiotics and adds complexity to our understanding of urosepsis risk factors [8–10].

The aim of this study is to provide a descriptive report of mortality and morbidity in the first 30 days after the diagnosis of urosepsis. We used the SIRS criteria, clinical findings, and microbiological confirmation of urinary tract infection as the underlying cause of sepsis. In addition, we aim to explore the risk factors for unfavourable outcomes expressed as organ failure and death by means of a comprehensive analysis of registered variables.

## Methods

This multi-center study assessed urosepsis in 34 European hospitals. Diagnosis required meeting at least two SIRS criteria, with the urinary tract identified as the sepsis source. Patients were followed for 30 days, with data collected at baseline and on days 3, 7, 9, and 30.

Sequential Organ Failure Assessment (SOFA) score domains were used to assess organ failure at predefined time points. Organ failure (OF) was defined as sustained impairment of normal organ function, hindering its physiological role. SOFA scores greater than 1 or an increase of 1 point from baseline indicated organ failure. Patients undergoing invasive supportive treatment (mechanical ventilation, vasopressor support, or renal replacement therapy) were also monitored at specified time points to track organ function.

### Study oversight and design

This prospective observational cohort study was conducted from September 2014 to November 2018 after ethical approval and registration. (Justus Liebig University, Giessen, Germany Ethical Board (AZ: 77/14) on 15/05/2014) (NCT02380170). The study, conducted from September 2014 to November 2018, was a prospective observational cohort study. It was approved and registered by the Justus Liebig University in Germany. Recruitment was monitored biweekly, and issues related to recruitment were managed between site investigators and the study management group (TEBJ, FW and ZT). The centers were selected from the Global Prevalence of Infections in Urology study [11].

Adult patients (≥ 18 years) diagnosed with acute urosepsis according to Sepsis-2 criteria were included in the study [12]. Patients were recruited from various healthcare settings, including emergency units, urology wards, other wards, outpatient clinics, and community care referrals. Urinary tract infection was confirmed through positive urine and blood cultures before antibiotic treatment. Patients with sepsis from other sites were excluded. Enrolled patients were categorized as non-severe or severe sepsis, including septic shock, based on Sepsis-2 definitions. Further study design details can be found in Supplement II [12].

### Data collection

Data were collected using an online case report form, with patient characteristics and physiological variables found in Supplement-III. SOFA items and additional clinical findings were gathered at diagnosis and follow-ups. Initial treatment details were recorded within the first 24 h of urosepsis onset, and follow-up assessments included outcomes and treatments. Data collection semi-automatic controlled, with any inconsistencies resolved by SMG to determine CRF eligibility (supplement IV).

Causative pathogens and their susceptibility profile were identified according to local practice, which included Clinical & Laboratory Standards Institute (CLSI) or European Committee on Antimicrobial Susceptibility Testing (EUCAST) criteria [13, 14]. Pathogens were classified as MDR or extensively drug-resistant according to the European Centre for Disease Prevention and Control (ECDC) and Centers for Disease Control and Prevention (CDC) joint initiative definitions [15].

### Statistical analysis

Anonymized data were analysed using the statistical package “R” version 3.2. Descriptive analysis summarized key information, including patient demographics, clinical variables, mortality rates, and rates of organ failure as a measure of morbidity. OF was categorized into single organ failure and multiple organ failure (MOF). The outcomes were one of four health states: death, MOF, single organ failure, and recovery.

Ordinal logistic regression analysis was used to evaluate the relationship between each risk factor and outcomes. A comprehensive analysis was conducted, incorporating all measured risk factors into the model, assessing the combined influence of these factors on outcomes.

## Results

### Diagnostic criteria and initial management

*Patient population*. The analysis included 354 patients meeting sepsis-2 criteria with identified pathogens (see patient case disposition in Supplementary Fig. 4) and completed 30-day follow-up. Median age was 65.1 years (IQR 51.1–74.1), with 45% females (*n* = 183). See Table 1 for patient details.

**Table 1:** Patient demographics and outcome on day 30

Characteristics			Urosepsis patients'	Day 30 outcome				
				Recovered	Single organ failure	Multiple organ failure	Death	*p*
			354	73.1% (259)	18.9% (67)	5.1% (18)	2.8% (10)	
Number of SIRS criteria	Sepsis		51% (181)	82% (149)	14%(26)	2% (4)	1%(2)	**0.00**
	Severe sepsis or septic shock		49% (173)	64%(110)	24%(41)	8%(14)	5%(8)	
Sex	Female		45% (158)	**75% (118)**	**17%(27)**	**5%(8)**	**3% (5)**	**0.91**
	Male		55% (196)	73% (143)	18% (35)	7% (13)	3% (5)	
Age	Mean (SD)		61.2 (16.8)	60.3 (17.4)	64.1 (15.1)	67.3 (10.7)	76.4 (12.3)	**0.00**
BMI	Mean (SD)		**26.6 (5.5)**	26.8 (6.0)	26.4 (4.2)	24.9 (2.9)	27.2 (3.5)	0.48
Charlson comorbidity index score	0		41% (146)	80% (117)	16% (23)	4% (6)	**0**	**0.00**
	1		19% (69)	71%(49)	23% (16)	6%(4)	0	
	2		18% (65)	71% (46)	22%(14)	6% (4)	0	
	> 2		21% (74)	66% (49)	12% (9)	9% (7)	12% (9)	
Long-term steroid treatment prior to urosepsis			4% (13)	46% (6)	31% (4)	8% (1)	**15%(2)**	**0.02**
Admission to intensive care unit due to an infection during the past 12 months			13% (47)	60% (28)	26% (12)	11% (5)	4% (2)	0.12
Previous genitourinary infection (past 12 months)	Any genitourinary infection episode		45% (125)	69% (86)	20% (25)	9% (11)	2% (3)	**0.05**
	Number of previous UTI episodes	0	53% (153)	82% (126)	15% (23)	2% (3)	1%(1)	**0.00**
		1 episode	22% (62)	79% (49)	11% (7)	8% (5)	2% (1)	
		≥ 2 episodes	23% (63)	59% (37)	29% (18)	10% (6)	3% (2)	
	Previous UTI condition	Cystitis	22% (60)	73% (44)	20% (12)	3% (2)	3% (2)	0.31
		Pyelonephritis	23% (64)	67% (43)	22% (14)	9% (6)	2% (1)	0.11
		Urosepsis	10% (27)	63%(17)	22% (6)	15% (4)	0	0.55
		Orchitis	3% (9)	56% (5)	33% (3)	11% (1)	0	0.55
		Prostatitis	5% (15)	93% (14)	7% (1)	0	0	0.34
Urinary tract obstruction at the time of diagnosis			% (174)	% (122)	% (36)	% (11)	% (5)	**0.04**
Urolithiasis	Any stone		42% (147)	75% (110)	18% (27)	5% (8)	1% (2)	0.54
	Stone location	Renal calyx	10% (35)	69% (24)	26% (9)	3% (1)	3% (1)	0.52
		Renal pelvis	14% (49)	67% (33)	27% (13)	2% (1)	4% (2)	0.19
		Ureter	27% (94)	77% (72)	16% (15)	6% (6)	1% (1)	0.62
		Bladder	4% (13)	77% (10)	8% (1)	15% (2)	0	0.36
	Number of locations	Nil	58% (207)	73% (151)	17% (35)	6% (13)	4% (8)	0.66
		Single	21% (109)	76% (83)	17%(18)	6% (7)	1% (1)	
		Multiple	11% (38)	71% (27)	24% (9)	3% (1)	3% (1)	
Antibiotic usage (past 3 months)			46% (128)	72% (92)	19% (24)	8% (10)	2% (2)	0.59
Hospitalization within the past 6 months			42% (204)	**70% (143)**	18% (37)	9% (18)	**3%(6)**	**0.04**
In-situ catheter at the time of diagnosis	Any catheter		42% (148)	**68%(100)**	17% (25)	10% (15)	**5% (8)**	**0.00**
	Number of catheters	1	32% (113)	**64% (72)**	18% (20)	12% (14)	6% (7)	**0.06**
		Multiple	10% (36)	81% (29)	14% (5)	3% (1)	3% (1)	
	Total catheter days**	< 2 days	14% (49)	71% (35)	16% (8)	6% (3)	**6%(3)**	**0.07**
		> 2 days < 30 days	20% (70)	64% (45)	20% (14)	11% (8)	4% (3)	
		> 30 days	8% (29)	69%(20)	10%(3)	14%(4)	7%(2)	
	Location	Urethral	20% (71)	61% (43)	20% (14)	10% (7)	10% (7)	**0.00**
		Suprapubic	3% (9)	67% (6)	33% (3)	0	0	0.54
		Nephrostomy	8% (30)	77% (23)	13% (4)	7% (2)	3% (1)	0.93
		Ureteral	13% (46)	74% (34)	13% (6)	11% (5)	2% (1)	0.41
	No catheters		58% (206)	78%	18%	3%	1%	
Previous urinary tract intervention		+	45% (159)	71% (113)	17% (27)	9% (14)	3%(5)	0.29
Health care associated infection		HAI	37% (131)	73% (96)	16% (12)	9% (21)	**2% (2)**	**0.81**
Urosepsis onset location	Hospital		54% ( 190)	35%(123)	13%(46)	5%(16)	1%(5)	**0.00**
	Community		46% (164)	39%(138)	5%(16)	1%(5)	1%(5)	

Bold indicates statistically singificant difference

#### Clinical presentation

3.4% of patients (*n* = 12) presented with septic shock, while 45.5% (*n* = 161) had severe sepsis. These two groups were combined and categorized as severe sepsis (*n* = 173), with the remaining cases classified as non-severe sepsis (*n* = 181). Additional information regarding baseline SIRS criteria is available in the supplementary materials (Table 1 in the Supplement).

#### Microbiological findings

Positive urine cultures were obtained in 338 patients, while positive blood cultures were obtained in 189 patients. Gram-negative bacteria were the predominant pathogens, accounting for 82% of urine cultures and 56.6% of blood cultures. S-Table 2 details pathogen frequencies in different cultures. MDR pathogens were observed in 28.5% of urine cultures and 21% of blood cultures (Fig. 5 in Supplement).

#### Initial management

All patients received antibiotics, either as a single agent (70%) or in combination (30%). The median time from diagnosis to the first antibiotic dose was 60 min (IQR 15–180 min). Antibiotic susceptibility data were available for 73% of cases (*n* = 260), and in 89% of these cases (*n* = 233), the causative pathogens were sensitive to the administered antibiotics. Urinary tract obstruction was relieved in 39.8% of patients (*n* = 141), with 72% of these cases (*n* = 101) addressed within 24 h of urosepsis diagnosis. Infected tissue debridement was performed in 6.5% of patients (*n* = 23), and among them, 56.5% (*n* = 13) underwent the procedure within the first 3 days after diagnosis.

### Mortality and organ failure

#### Mortality

Mortality rate within 30 days was 2.8% (*n* 10). Mean time to death from the point of diagnosis of urosepsis was 8.4 days (min 3.6–max 25.2). All patients who died presented with OF at diagnosis. The mortality rate was higher in patients who presented with kidney (5% vs no kidney failure: 0, *p* = 0.01), respiratory (8% vs no respiratory failure: 1%, *p* = 0.00), and cardiovascular system failure (7% vs no failure: 2%, *p* = 0.04).

#### Dynamics of organ failure

**Fig. 1:**
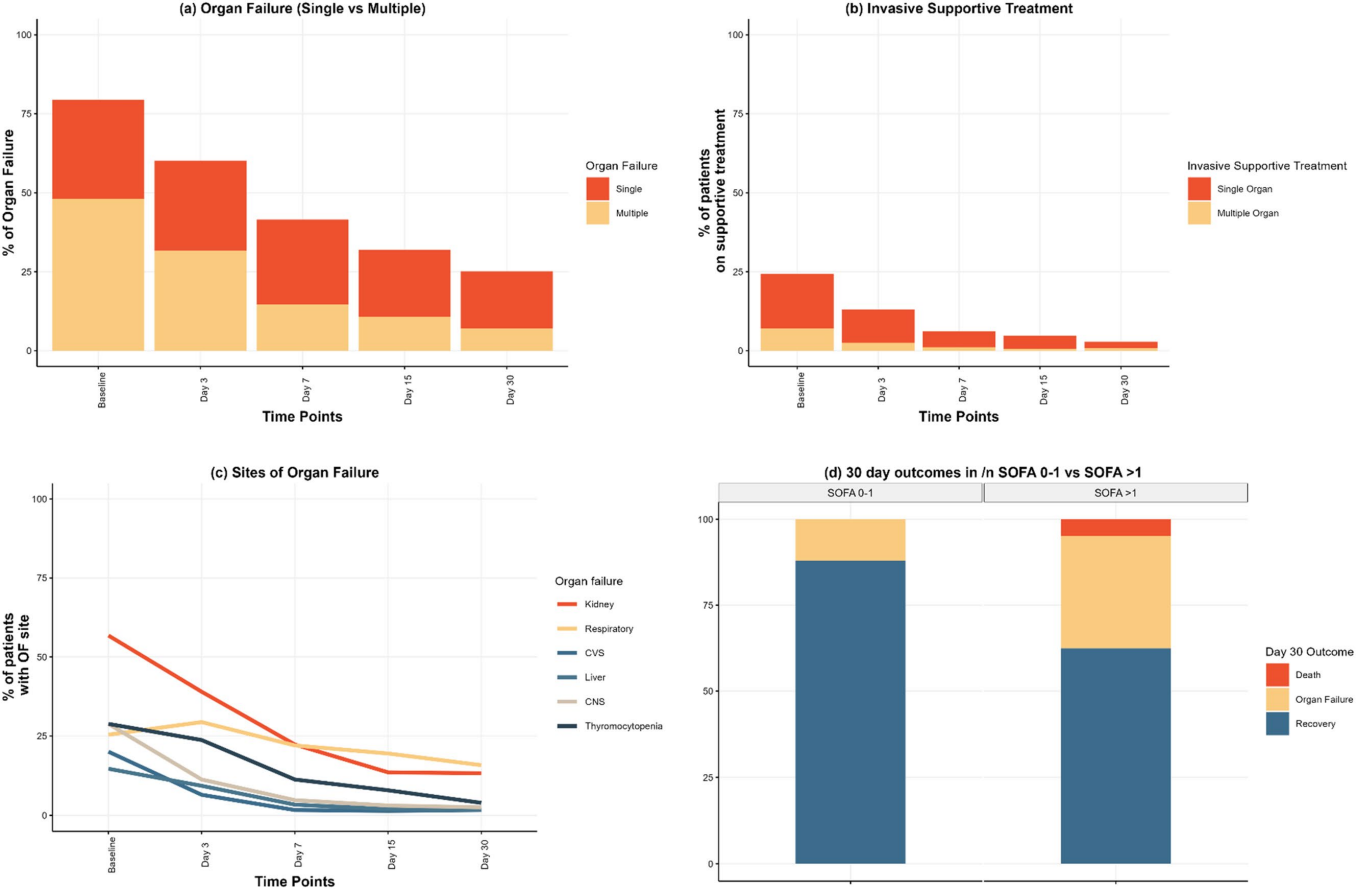
Details of organ failure over 30 days follow-up. **a** Proportion of patients with multiple organ failure. **b** Proportion of patients that require invasive supportive treatment and (**c**) proportion of patients with sites affected. **c** Among the cases of OF, kidney failure (red line) was most common at the time of diagnosis (57%) until day 3 (37%). However, by day 7, both kidney (22%) and respiratory failure (yellow line) (22%) were the leading causes. This trend persisted on day 15 (kidney: 13% and respiratory: 19%) and day 30 (kidney: 13% and respiratory: 16%), where respiratory failure surpassed kidney failure as the predominant cause of OF

Initially, 79% (*n* = 281) of patients developed OF, with an additional 5% developing within 30 days, affecting 84%. The rate decreased to 24% (*n* = 85) by the end of the 30-day period. Initially, 48% (*n* = 170) of the cohort had MOF, which decreased to 7% (*n* = 25) on day 30 (Fig. 1a, b). Time trends of the OF sites are explained in Fig. 1c.

#### Impact of comorbidity and risk factors

Sepsis severity was greater in patients who died or had OF on day 30 (*p* = 0.00). Details of individual variables and their distribution among outcome groups at day 30 are available in Table 1. Among patients with MDR pathogens detected in either urine or blood cultures, 33% (*n* = 31) had organ failure at day 30, while only 20% (*n* = 52) of patients without MDR pathogens exhibited organ failure (*p* = 0.03).

The logistic ordinal regression analysis populated with all risk factors identified Charlson score (OR 1.16 CI 1.03–1.30), patients with respiratory failure at baseline (OR 3.50, CI 2.01–6.12), history of UTIs and its frequency (OR 1.74, CI 1.11–2.74), urosepsis with MDR pathogens (OR 1.66, CI 1.12–2.74), and sepsis severity (OR 2.33, CI 1.38–3.95) are significantly associated with day 30 outcomes (organ failure and death).

#### Impact of SOFA score

A total of 200 (56%) patients had a SOFA score of ≥ 2 at diagnosis and patients who died were all within this group. SOFA score of ≥ 2 at diagnosis was significantly associated with both single OF and MOF at day 30 (*p* = 0.00) (OR 4.38, CI 2.48–7.74) (Table 2).

**Table 2:** Outcomes of patients at day 30 with baseline SOFA score < 2 vs ≥ 2

	Recovered	Single organ failure	Multiple organ failure	Death	*p*
All patients	73.1% (259)	18.9% (67)	5.1% (18)	2.8% (10)	
Baseline SOFA < 2	87%(125)	11.9%(17)	0.7%(1)	0	0.00
Baseline SOFA ≥ 2	62.5% (125)	25% (50)	8%(16)	4.5% (9)	

The logistic ordinal regression analysis repeated with SOFA baseline categories instead of SIRS severity, indicates that Charlson score (OR 1.14, CI 1.01–1.28), patients with respiratory failure at baseline (OR 2.35, CI 1.32–4.21), ICU admission within the past 12 months (OR 2.05, CI 1.00–4.19), obstruction causative of urosepsis (OR 1.76, CI 1.02–3.05), urosepsis with MDR pathogens (OR 2.01, CI 1.15–3.53) and SOFA baseline score ≥ 2 (OR 2.74, CI 1.49–5.07) are significantly associated with day 30 outcomes (OF and death).

## Discussion

Our study aimed to investigate the mortality and morbidity outcomes of urosepsis and identify important risk factors associated with them.

### Main findings

The 30-day mortality rate for urosepsis meeting SIRS criteria and confirmed microbiologically was 2.8%, rising to 4.6% for severe sepsis. Higher Charlson scores, respiratory failure at urosepsis diagnosis, and urosepsis caused by MDR pathogens were associated with ongoing OF after 30 days, increased severity of OF, and higher mortality rates. Patients with recent health events (ICU admissions, UTIs) and higher UTI frequency had a greater risk of OF at 30 days. Urinary tract obstruction was also related to negative outcomes. A baseline SOFA score ≥ 2 was a significant predictor of death, with all deaths occurring within this group.

### Findings compared with other studies

The sepsis mortality rate varies, commonly reported around 10%, depending on factors like sepsis source, population studied, local AMR prevalence, and management protocols [1–4]. However, our study found a lower mortality rate due to lower proportion of severe sepsis cases, a favourable Charlson score (0–1) in 60% of the cohort and timely management [16, 17]. Still, severe sepsis cases had a notable 4.6% mortality rate.

### The importance of risk factors

Studies show AMR's potential to increase sepsis mortality at the population level [8, 10, 18]. For example, a recent study suggests AMR's association with pyelonephritis progressing to sepsis [18]. Our findings also indicate MDR pathogens as significant predictors of urosepsis mortality and organ failure. In a Swedish retrospective study community onset urosepsis with blood stream infection (2019 and 2020), low AMR rates were observed and inappropriate empirical treatment was associated with mortality [16]. In our study in 89% of cases, the pathogens were susceptible to the antibiotics given. However, achieving a high rate of appropriate antibiotic administration involved the use of reserve antibiotics. Specifically, 25% of patients (*n*: 87) received Carbapenem group antibiotics (results not presented).

We found that, a history of UTI within the past 12 months increased the risk of mortality and morbidity, potentially due to the presence of MDR pathogens in persistent infection sites. Further analysis revealed higher rates of MDR pathogens in patients with UTI history (37%—*n* = 56) compared to those without (19%, *n* = 38; *p* = 0.00). Similar patterns were observed for other risk factors, such as previous ICU admission (49% MDR rate vs. 23% without admission) and the presence of indwelling catheters (35% MDR rate vs. 21% without catheter). However, poorer outcomes cannot be solely attributed to MDR pathogens as factors like impaired immune responses, frailty, and functional disorders may also contribute [19]. The complex pathogenesis of urosepsis necessitates a differentiated management approach, with consideration of urological risk in guiding empirical treatment.

### Clinical implications

All deaths in our study were observed exclusively in patients with a baseline SOFA score of ≥ 2, leading to a mortality rate of 5% within this specific subgroup. Furthermore, our research reveals that 62.4% of patients with a SOFA < 2 experienced progression to develop OF, with 13% still experiencing OF at day 30. (s-Table 3). This is in comparison to 33% of patients with a SOFA ≥ 2 (Table 2). Our study supports the use of the SOFA score in confirming the diagnosis of urosepsis. It highlights that timely identification and immediate intervention result in improved results, underscoring the significance of further evaluation for risk classification, escalating or de-escalating treatment.

### Weaknesses

The SERPENS study, which was carried out by urology teams, was initially powered by an anticipated mortality rate of over 10%. Nevertheless, the death rate recorded in the research was considerably lower, suggesting that the study may have lacked sufficient statistical power. The observed phenomenon can be attributed to a selection bias that impacted by discrepancies in patient treatment among diverse healthcare jurisdictions.

### Methodology

In our study, we used ordinal categories to measure mortality and organ dysfunction severity, with a potential alternative methodology using organ failure-free days [20]. This method provides a comprehensive understanding of organ functionality and quantitative evaluation of urosepsis' influence on organ failure and recovery. Future research could use this methodology to gain additional perspectives on urosepsis outcomes.

## Conclusions

This study provides new information about the mortality and morbidity patterns associated with contemporary urosepsis. Analysis of risk factors emphasizes the impact of comorbidities and MDR pathogens on outcomes and highlights the existence of a distinct group of patients who do not initially present with organ failure but may progress to a more severe state. These findings underscore the need for the development of pragmatic classifications to better assess the severity of UTIs and guide management strategies.

## Supplementary Information

Below is the link to the electronic supplementary material.

Supplementary file1 (DOCX 486 KB)

## Data Availability

Data will be made available upon request to the corresponding author and will be subject to approval by the study coordinators for research purposes.
