# Supplementary material for: Urosepsis 30-day mortality, morbidity, and their risk factors: SERPENS study, a prospective, observational multi-center study
Source: World J Urol. 2024 May 10;42(1):314. doi: 10.1007/s00345-024-04979-2 (PMC11087335; doi:10.1007/s00345-024-04979-2)
Supplement: Supplementary file 1 — Supplementary file1 (DOCX 486 KB) [file 345_2024_4979_MOESM1_ESM.docx]

Contents

[1 Inclusion and Exclusion Criteria 2](#_Toc154879695)

[2 Study Design 3](#_Toc154879696)

[3 Study measures 5](#_Toc154879697)

[4 Data Check and Validation 8](#_Toc154879698)

[4.1 Automated check 8](#_Toc154879699)

[4.2 Manual Check 8](#_Toc154879700)

[4.3 Case-by-case checks 8](#_Toc154879701)

[5 Missing data 10](#_Toc154879702)

[6 Patient Case Disposition 11](#_Toc154879703)

[7 Further Results 12](#_Toc154879704)

[7.1 Sepsis Criteria 12](#_Toc154879705)

[7.2 Microbiological findings 13](#_Toc154879706)

[7.3 MDR Pathogens in urosepsis 13](#_Toc154879707)

[7.4 Urosepsis Outcomes 14](#_Toc154879708)

[7.4.1 Risk factors of outcomes adjusted for sepsis severity and their relationship with MDR bacteria in urine 18](#_Toc154879709)

[8 SERPENS Study Investigators 19](#_Toc154879710)

# Inclusion and Exclusion Criteria

Inclusion Criteria:

- Microbiologically proven infection in the urogenital tract
- Age>18
- Patients must meet at least two of the following four SIRS criteria, at least one of which must be the core temperature criterion or the WBC criterion; these criteria did not have to be met simultaneously:
  1. Hypothermia by core temperature <36ºC, or hyperthermia >38ºC measured via any means
  2. Heart rate (HR) >90 beats per minute
  3. Respiratory rate (RR) >20 breaths/minute related to septic event, or partial pressure of arterial carbon dioxide (PaCO2) <32 mmHg related to septic event or requiring mechanical ventilation related to septic event
  4. Total WBC absolute count >12,000 cells/mm^3^ or <4000 cells/^mm3^

Exclusion Criteria:

- Sepsis due to other causes outside the urogenital tract

# Study Design

Patients with sepsis were screened for the urinary tract as the source of infection based on established clinical criteria. Clinical criteria used to establish suspicion for the urinary tract as the source of infection included;

- Recent genitourinary tract procedures (0-48 hours)
- Indwelling urinary catheters
- New onset clinical symptoms and signs (anyone):
  - Flank pain
  - Dysuria
  - Frequency
  - Urgency
  - Genital or suprapubic pain
  - Costovertebral tenderness

**s-Figure 1. Patient recruitment to SERPENS study.** Patients with a confirmed diagnosis of sepsis were screened for suspicion of the urinary tract (clinical criteria) as the source of infection. If proven, patients were recruited to SERPENS.


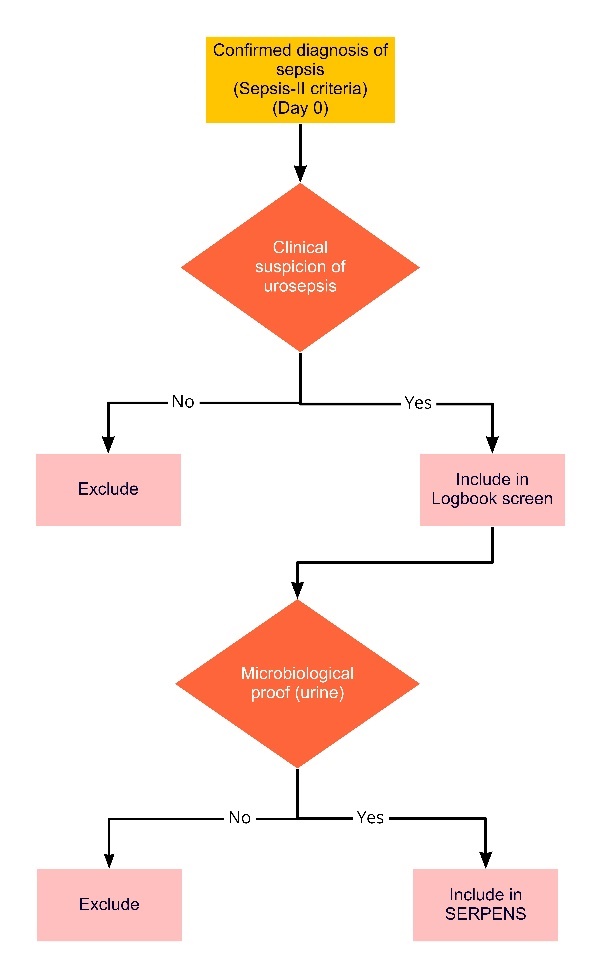


Initial screen to enter the study was carried out on day 0 when suspicion of urosepsis was reported. All patients that fulfilled the eligibility criteria (Supplement-I) were included in the SERPENS study (s-Figure 2).

**s-Figure 2. Prospective observational cohort of urosepsis patients.** All patients with suspicion of urosepsis were followed for 30 days and data collection was carried out at five predefined time points (day 0, 3, 7, 9 and 30). Patients with confirmed urosepsis diagnosis (microbiological proof of infection) were included in SERPENS study.


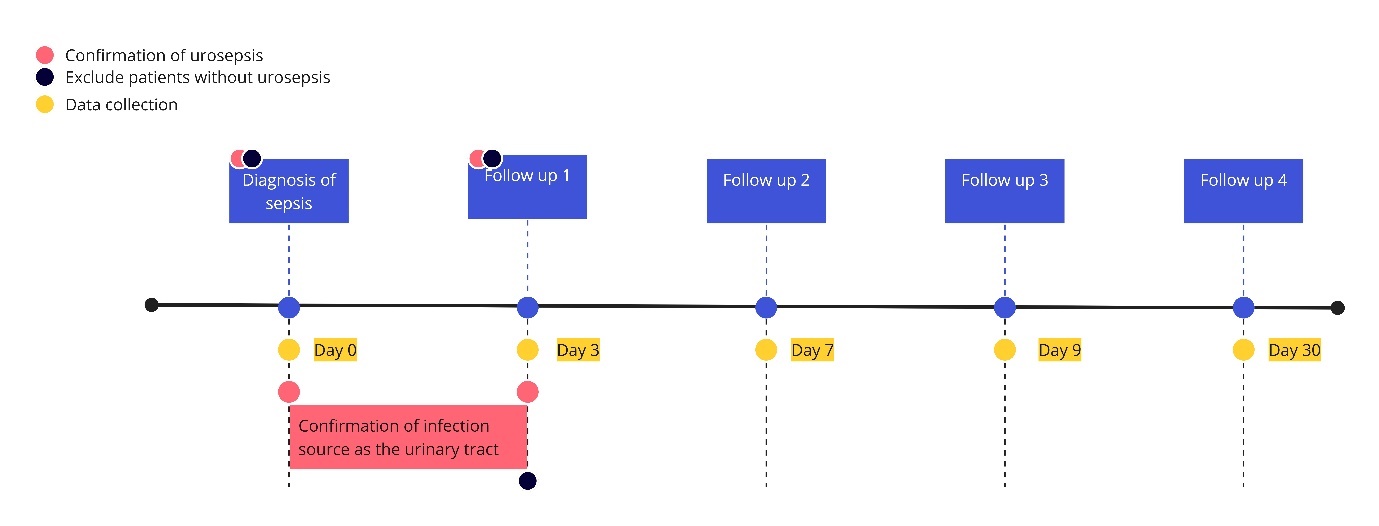


Site selection and quality assurance of sites:

Participating centres were part of the Global Prevalence of Infections in Urology (GPIU) study initiative, which is an annual study conducting point prevalence studies of infections in urology departments ^1^. Sites were selected based on consistent and high recruitment in the GPIU study. Site investigators were trained and familiarized with the study protocol by the SMG. Regular group meetings were held between the SMG and site investigators to update on recruitment and address any issues. Based on this feedback a protocol amendment was carried out in July 2015 when a health economic assessment form was made optional. Consecutive regular quality assurance meetings continued until recruitment was completed.

# Study measures

Patient baseline variables:

- Age
- Body weight
- Height
- Body mass index
- Date and time of admission to hospital
- Reason for admission to hospital
  - Urosepsis
  - Urinary tract infection
  - Urological condition
  - Other medical condition
- Where was the patient admitted from:
  - home
  - nursing home
  - another hospital
  - outpatient clinic
  - emergency department
  - another department in the same hospital
- Presenting signs and symptoms of urinary tract infection
  - Costovertebral tenderness
  - Dysuria, Frequency, urgency
  - Flank pain
  - Genital pain
  - Others
- SIRS criteria
- SOFA criteria
- Organ failure at diagnosis
- ORENUC (risk assessment tool for UTIs)

Patient risk factors:

- Medical risk factors
  - Charlson comorbidity index items
  - Charlson score
  - Karnofsky performance score
  - Steroid usage for other conditions
  - Previous admission to intensive care unit within past 12 months due to infections
- Urological risk factors
  - History of UTIs
    - Site
    - Number
  - Urinary tract obstruction
    - Location within genitourinary tract
    - Cause of obstruction
    - Unilateral or bilateral
    - Was the obstruction likely cause of the episode of urosepsis
  - Urinary tract stones
    - Location
    - Number
  - Recent antibiotic treatment (3 months)
    - Reason
    - Number
  - Hospital admission within past 6 months
  - Indwelling urinary catheters
    - Type (urethral, nephrostomy, ureteric, suprapubic)
    - Number
    - Duration
  - Previous urinary tract interventions
    - Time
    - Type of intervention
      - ICD codes
      - Open vs Endoscopic vs Minimally invasive
      - Contamination
    - Complications
      - Clavien Dindo grade
- Microbiology
  - Urine culture
  - Blood culture
  - Other sites
  - Causative primary and secondary pathogen in specimen
  - Antibiotic susceptibility profile
- Initial Management (first 24 hours)
  - Antibiotic treatment
    - Number
    - Time of administration
    - Name of antibiotic/s
    - Dose
  - Medical supportive treatment
    - Ventilation support (Non-invasive, Mechanical Intubation, Tracheostomy)
    - Blood product administration (Fresh frozen plasma, Erythropoietin, Antithrombin administration, Platelets)
    - Renal failure support (Haemodialysis, Hemofiltration, Peritoneal dialysis)
    - Hemodynamic support and adjunctive therapy (Vasopressors: Norepinephrin, Epinephrin, Vasopressin, Dopamine, Phenylephrine, Corticosteroids, Others)
    - Intravenous immunoglobulins
    - Coagulative system support
    - Hepatic failure/dysfunction support:
    - Endocrinological failure/dysfunction support:
- Further management measured up to 30 days:
  - EQ-5D (removed after protocol review)
  - SOFA score
  - New onset organ failure (Kidney, cardiovascular system, pulmonary, hepatic, ileus, thrombocytopenia) classified using the SOFA score
  - Degree of recovery of organ failure (I.e. complete recovery: back to baseline, partial improvement: recovery observed but still not back to baseline)
  - Supportive treatment provided for each organ (as measured in Initial management medical supportive treatment)
  - Clinical status based on SIRS (no sepsis, sepsis, severe sepsis, septic shock)
  - Death
  - Treatment unit (ICU, urology ward, internal medicine ward)
  - Change in antibiotics
    - Date
    - Numbers
    - Antibiotic/s
  - Urological interventions to manage the source
    - Date
    - ICD code
    - Decompressive intervention, removal of infected tissue
    - Complications (Clavien Dindo)
  - Change in medical supportive treatment (new, weaning, stop)

# Data Check and Validation

Data validity was ensured by implementing multiple checks at different points before approval for the CRF to be registered on the database. These included;

1. *automatic check* of the online CRF to identify logical errors,
2. *manual check* of logical errors, case inconsistencies and inconsistencies per case vs. site, other sites and already established knowledge
3. *case by case check* of any persistent errors not resolved with automatic and manual checks

The process of data validation is illustrated in **s-Figure 3. Data validation and check process map.**

## Automated check

The online CRF was built in with logical check arguments. These included date and time check of diagnosis, interventions and outcomes recorded. For instance, if a patient was recorded to have sepsis on day 0 and the initial administered antibiotic was registered over 24 hours this would be picked up. All entered dates and times within the CRF were subject to similar check where logical “if” scenarios were built in by the principal investigators.

Any errors flagged up by the site investigator that could not exit this loop were picked up on the 3^rd^ error loop and brought to the attention of the study managers. These were dealt with on a case-by-case scenario and direct contact was made to site investigators. Cases that remained unresolved were excluded. Data identified as valid in the automatic check would proceed to the manual check.

## Manual Check

Manual check was carried out by the data manager and clinical study PI (ZT). At regular intervals, each case was analysed to identify case-based inconsistencies. If the cases were identified as valid, they were registered in the final database.

Manual check consisted of two steps.

Step 1 consisted of mapping of the case journey from diagnosis to day 30 follow-up. This data was reviewed by the clinical principal investigators (ZT, FW, TEBJ). Any clinical uncommon practices, outcomes or potential errors were highlighted. These were communicated with the site investigator to understand if this was an entry error, shortfall of the CRF or invalid case. After final information was gathered for cases with flagged issues this would be discussed amongst the clinical principal investigators (ZT, FW, TEBJ) to identify validity.

Step 2 consisted of analysing the pooled interim data for interventions and outcomes (pathogens, susceptibility profile, organ failure and death). Outlying cases were identified and would be subject to review and discussion between the site investigator and clinical principal investigators. This would ensure any inadvertent errors in data entry.

CRFs that passed step 1 and 2 were registered in the final database and locked.

## Case-by-case checks

As highlighted in previous steps cases unresolved issues with automatic checks, case-based inconsistencies by manual check and outliers from pooled analysis were subject to case-by-case review.

The process involved contacting the site investigators. Site visits were not necessary and instead online meetings were carried out with the site investigator. If case-based inconsistencies were not resolved they would be excluded.

**s-Figure 3. Data validation and check process map.**


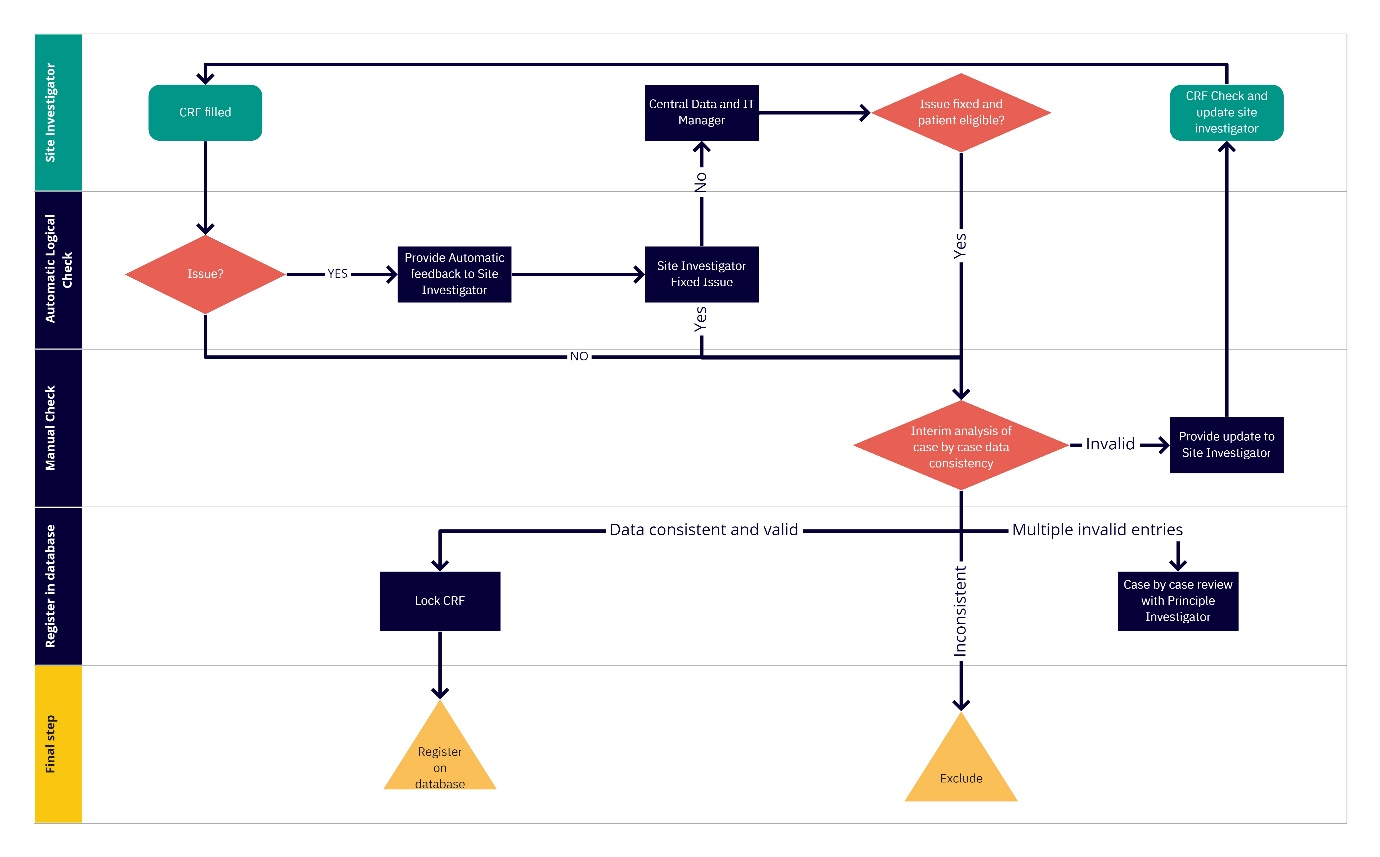


# Missing data

Under the assumption of missing data at random, missing data in confounders was addressed by applying imputation using linear regression for continuous variables and logistic regression for dichotomous variable. Sensitivity analysis was conducted by checking the impact of imputation on outcomes of the modelling. This was achieved by obtaining the outcomes with three different imputation methods that included imputation by the predicted mean, lower and upper confidence intervals. Three sets of analyses were conducted on the outcomes and in the paper the mean values of imputation are presented. The sensitivity analysis with different imputation values revealed no difference in outcomes estimated through multinomial regression modelling.

# Patient Case Disposition

The initial population with suspicion of urosepsis consisted of 537 patients. Of these 125 exhibited no growth on cultures or missing culture information. Of the 412 patients with culture proven urosepsis 58 patients with protocol violations and missing follow-up information were excluded from final analysis. This is summarized in s-Figure 4.

**s-Figure 4.** Patient case disposition of SERPENS study.


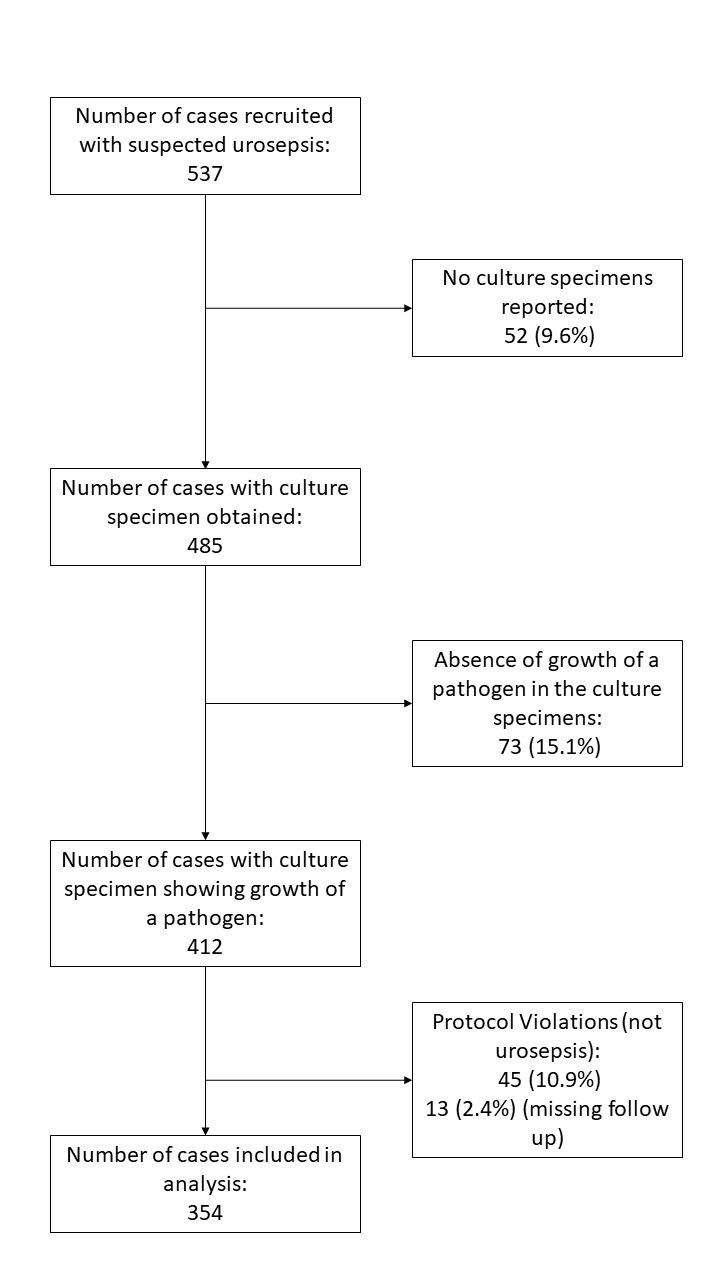


# Further Results

## Sepsis Criteria

s-table 1. Frequency of SIRS criteria at initial diagnosis of urosepsis in the study population.

| Heart rate | | |
| --- | --- | --- |
| **<90** | 19.8% (n:70) | |
| **>90** | 79.4% (n:281) | |
| **Missing** | 0.8% (n:3) | |
| Body temperature | | |
| **<36^0^C** | 1.4% (n:5) | |
| **36-38^0^C** | 18.4% (n:65) | |
| **>38^0^C** | 79.7% (n:282) | |
| **Missing** | 0.6% (n:2) | |
| Respiratory Criteria | | |
| **Respiratory rate** | <20 | 39.5% (n:140) |
|  | >20 | 57.9% (n:205) |
|  | Unreported | 2.5% (n:9) |
| **PaCO_2_** | <32mmHg | 18.9% (n:67) |
|  | >32mmHg | 64.1% (n:227) |
|  | Unreported | 16.9% (n:60) |
| **Mechanical Ventilation** | Yes | 4.5% (n:16) |
|  | No | 92.9% (n:329) |
|  | Unreported | 2.5% (n:9) |
| **At least one respiratory criterion for SIRS present** | Yes | 64.4% (n:228) |
|  | No | 35.6% (n:126) |
| White blood cell count | | |
| **<4.000 cells/mm^3^** | 5.1% (n:18) | |
| **4.000-12.000 cells/mm^3^** | 18.6% (n:66) | |
| **>12.000 cells/mm^3^** | 75.4% (n:267) | |
| **Missing** | 0.8% ( n:3) | |

## Microbiological findings

**s-table 2.** Pathogens identified in patients with urosepsis. Both a positive urine and blood culture were detected in 174 (51%) patients. In all patients with culture specimens obtained from other sites either the urine (n=16) or blood (n=6) culture resul

| Gram staining | | **Urine** | **Blood** | **Other sites** |
| --- | --- | --- | --- | --- |
| **Gram negative** | **E. coli** | 50.9% (n:172) | 29.1% (n:55) | 5.3% (n:1) |
|  | **Klebsiella sp.** | 13.9% (n:47) | 12.7% (n:24) | 21.1% (n:4) |
|  | **Proteus sp.** | 6.2% (n:21) | 8.5% (n:16) | 21.1% (n:4) |
|  | **P. aeruginosa** | 3.6% (n:12) | 1.6% (n:3) | 5.3% (n:1) |
|  | **Enterobacter sp.** | 3.8% (n:13) | 3.7% (n:7) | 0 |
|  | **Acinetobacter sp.** | 1.2% (n:4) | 0 | 5.3% (n:1) |
|  | **Other Enterobacteriaceae** | 1.2% (n:4) | 0.5% (n:1) | 0 |
|  | **Morganella sp.** | 0.9% (n:3) | 0.5% (n:1) | 5.3% (n:1) |
|  | **Citrobacter sp.** | 0.3% (n:1) | 0 | 0 |
|  | **Gram negatives** | 82% (n:277) | 56.6% (n:107) | 63.2% (n:12) |
| **Gram positive** | **Enterococcus sp.** | 11.1% (n:38) | 5.3% (n:10) | 5.3% (n:1) |
|  | **CNS** | 0 | 5.8% (n:11) | 5.3% (n:1) |
|  | **Staphylococcus aureus** | 0.9% (n:3) | 3.2% (n:6) | 5.3% (n:1) |
|  | **Other Gram (+) cocci** | 1.5% (n:5) | 2.6% (n:5) | 5.3% (n:1) |
|  | **Gram positives** | 13.5% (n:46) | 16.9% (n:32) | 21.1% (n:4) |
| **Other bacteria** | | 2.4% (n:8) | 1.6% (n:3) | 0 |
| **Fungi** | **Candida sp.** | 0.9% (n:3) | 2.1% (n:4) | 5.3% (n:1) |
| **No growth** | | 1.2% (n:4) | 22.8% (n:43) | 10.6% (n:2) |

## MDR Pathogens in urosepsis

s-Figure 5 MDR pathogens identified in urine and blood samples. Of the urine pathogens 28.5% (88), 31.7% (98) and 39.8% (123) were MDR (resistant to ≥1 agent in ≥3 antimicrobial categories), resistant to ≥1 agent in <3 antimicrobial categories and susceptible to all tested antibiotics (indicated with grey). From blood culture specimens none exhibited features of pan-susceptibility and 21% (n: 28) were MDR. (MDR= The isolate is non‐susceptible to at least 1 agent in ≥3 antimicrobial categories)(3). Pathogens without any resistance features (blue) were detected in 40% of urine cultures but there were none in blood cultures.


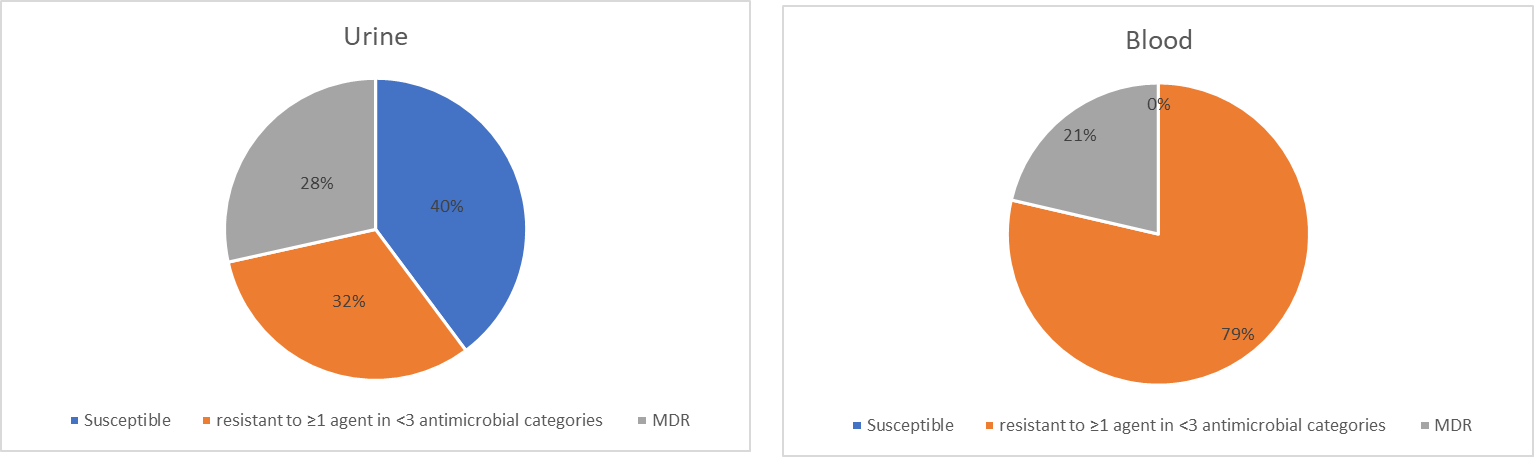


## Urosepsis Outcomes

s-table 3. Clinical outcomes from onset of urosepsis classified according to different sepsis tools.

|  | | **Overall Cohort**  **(354)** | **SIRS criteria severity** | | **SOFA score** | |
| --- | --- | --- | --- | --- | --- | --- |
|  |  |  | Sepsis (181) | Sever sepsis or septic shock (173) | <2  (149) | ≥2  (205) |
| **Outcomes on day 30** | Organ failure or death | 26.8% (95) | 17.7% (32) | 36.4% (63) | 12.1% (18) | 37.6% (77) |
|  | p | | 0.00 | | 0.00 | |
|  | Organ failure | 24.0% (85) | 16.6%(30) | 31.7%(55) | 12.1% (18) | 32.6% (67) |
|  | p | | 0.00 | | 0.00 | |
|  | MOF | 5.1%  (18) | 2.2%  (4) | 8.1%  (14) | 0.6%  (1) | 8.3%  (17) |
|  | p | | 0.02 | | 0.00 | |
|  | Supportive treatment | 1.1%  (4) | 0 | 2.3%  (4) | 0.6%  (1) | 1.5%  (3) |
|  | p | |  | |  | |
|  | Death | 2.8% (10) | 1.1% (2) | 4.6% (8) | 0 | 4.9% (10) |
|  | p | | 0.09 | | 0.02 | |
| **Organ failure within 30 days** | At least 1 organ | 84.2% (298) | 73.5% (133) | 95.4%  (165) | 62.4% (93) | 100% (205) |
|  | p | | 0.00 | | 0.00 | |
|  | MOF | 56.2% (199) | 40.9% (74) | 72.3%  (125) | 14.7% (22) | 86.3% (177) |
|  | p | | 0.00 | | 0.00 | |

s-Table 3. New onset organ failure, within 30-days of diagnosis and at day 30.

|  | | **At any time point** | **30 day course of organ impairment and failure** | | | | |
| --- | --- | --- | --- | --- | --- | --- | --- |
|  |  |  | Diagnosis  (n=354) | 3  (n=350) | 7  (n=350) | 9  (n=348) | 30  (n=344) |
| **Kidney** | Failure | 61.3 % (217) | 53.4%  (189) | 37.7% (132) | 22.3% (78) | 13.9%  (48) | 11.3%  (39) |
|  | Dialysis | 2.5%  (9) | 1.9%  (7) | 0.9%  (3) | 0.3%  (1) | 0.3%  (1) | 0.6%  (2) |
| **Respiratory** | Failure | 70.3 % (249) | 68.6% (243) | 30.6%  (107) | 22.3%  (78) | 19.8%  (69) | 15.7%  (54) |
|  | NIMV° | 14.4%  (51) | 7.9%  (28) | 7.4%  (26) | 4.0%  (14) | 3.4%  (12) | 0.6%  (2) |
|  | Intubated | 5.7%  (20) | 4.5%  (16) | 2%  (7) | 1.1%  (4) | 0.6%  (2) | 0.3%  (1) |
| **CVS** | Failure | 24.8% (88) | 20.9%  (74) | 10.3%  (36) | 2.6%  (9) | 2.0%  (7) | 2.0%  (7) |
|  | Inotropic treatment | 10.5%  (37) | 9.1%  (32) | 2.9%  (10) | 0.9%  (3) | 0.3%  (1) | 0.9%  (3) |
| **Liver impairment** | | 19.8% (70) | 15.3%  (55) | 9.4%  (33) | 3.4%  (12) | 2.0%  (7) | 1.7%  (6) |
| **CNS** | GCS** (<15) – total cases | 32.2% (114) | 28.8%  (102) | 11.4%  (40) | 4.9%  (17) | 3.2%  (11) | 2.6%  (9) |
|  | Mild -GCS** (13-14) | 28.5%  (101) | 24.9% (88) | 7.7%  (27) | 3.7%  (13) | 1.7%  (6) | 0.9%  (3) |
|  | Moderate - GCS* (10-12) | 5.4%  (19) | 3.4 % (12) | 2.0%  (7) | 0.6%  (2) | 0.9%  (3) | 0.3%  (1) |
|  | Severe - GCS** (≤9) | 3.4%  (12) | 0.6%  (2) | 1.7%  (6) | 0.6%  (2) | 0.6%  (2) | 1.5%  (5) |
| **Thrombocytopenia** | | 44.6% (158) | 36.4% (129) | 26.9%  (94) | 11.4%  (40) | 8.0%  (28) | 4.1%  (14) |
| **Ileus** | | 10.2%  (36) | 9.1%  (32) | 2.6%  (9) | 0.3%  (1) | 0.3%  (1) | 0.3%  (1) |
| **Organ failure** | Multiple (>1) | 72.6%  (257) | 50%  (177) | 28%  (99) | 14.8%  (52) | 10.0%  (35) | 5.8%  (20) |
|  | At least 1 | 86.2%  (305)Δ | 82.2%  (291) | 58.3%  (204) | 41.1% (144) | 32.8%  (114) | 23.8%  (82) |
| **Organ supportive treatment** | At least 1 | 20.3% (72)Ϫ | 17.2%  (61) | 10.0%  (35) | 5.1%  (18) | 4.0%  (14) | 1.2%  (4) |
|  | Multiple (>1) | 8.7%  (31) | 7.1%  (25) | 2.3%  (8) | 1.1% (4) | 0.5%  (2) | 0.3%  (1) |
| **Mean SOFA Score** |  |  | 4.8 (1-23) | 1 (0-2) | 0 (0-1) | 0 (0-1) | 0 (0-1) |

*Chi-square test. °NIMV: Non Invasive Mechanical Ventilation. **:GCS: Glasgow Coma Scale

Δ: In total 305 patients had at least one organ impairment. Of these patients 257 had multiple organs involved and 72 of them had an organ failure also. Of the 305 patients 10 died within 30 days after onset of urosepsis.

Ϫ: of the 72 patients who developed organ failure 7 died within 30 days after onset of urosepsis.

s-Table 4. Clinical outcomes of sepsis according to baseline variables.

|  |  | | Death | Organ  Failure or Mortality on day 30 | Organ failure | |
| --- | --- | --- | --- | --- | --- | --- |
|  |  | |  |  | On day 30 | Within 30 days |
| Sex | Female | | 3.2% (5) | 25.3% (40) | 22.2% (35) | 77.2% (122) |
|  | Male | | 2.6% (5) | 28.1% (55) | 25.5% (50) | 89.8% (176) |
|  | p | | 0.98 | 0.64 | 0.54 | 0.00 |
| Age | Mean (SD) | | 76.4 (12.3) | 61.8 (16.9) | 64.9 (14.4) | 63.8 (15.7) |
|  | p | | 0.00 | 0.00 | 0.04 | 0.00 |
| Body mass index | Mean (SD) | | 27.2 (3.8) | 26 (3.7) | 25.8 (3.7) | 26.5 (5.6) |
|  | p | | 0.74 | 0.18 | 0.13 | 0.38 |
| Charlson comorbidity index score | 0 | | 0 (0) | 19.9% (29) | 19.9% (29) | 74.7% (109) |
|  | 1 | | 0 (0) | 26.1% (18) | 26.1% (18) | 86.9% (60) |
|  | 2 | | 1.5% (1) | 29.2% (19) | 27.7% (18) | 96.9% (63) |
|  | >2 | | 12.2% (9) | 39.2% (29) | 27.0% (20) | 89.2% (66) |
|  | Mean (SD) | | 5.3 (2.1) | 2.3 (1.3) | 1.9 (2.4) | 1.7 (2.1) |
|  | p | | 0.00 | **0.00** | 0.08 | 0.00 |
| Long term Steroid Treatment prior to urosepsis | + | | 15.4% (2) | 63.6% (7) | 45.5% (5) | 100% (11) |
|  | - | | 2.3% (8) | 25.7% (88) | 23.3% (80) | 83.8% (287) |
|  | p | | 0.02 | **0.01** | 0.18 | 0.29 |
| Admission to intensive care unit due to an infection (past 12 months) | + | | 4.3% (2) | 42.5% (20) | 38.3% (18) | 82.9% (39) |
|  | - | | 2.6% (8) | 24.4% (75) | 21.8% (67) | 84.4% (259) |
|  | p | | 0.87 | **0.01** | **0.02** | 0.99 |
| Previous genitourinary infection (past 12 months) | | No previous UTI | 1.9%(6) | 21.9% (43) | 19.9% (37) | 84.8% (134) |
|  |  | At least 1 episode | 3.9%(4) | 32.9% (52) | 29.1% (48) | 83.7% (164) |
|  |  | p | 0.5 | **0.02** | **0.05** | 0.88 |
| Urinary tract obstruction at the time of diagnosis | Obstruction causative for the episode of urosepsis | Yes | 2.8%(5) | 29.9% (52) | 27.0% (47) | 87.9% (153) |
|  |  | No | 2.8%(5) | 23.9% (43) | 21.1% (38) | 80.6% (145) |
|  |  | p | 1 | 0.24 | 0.24 | 0.07 |
| Urolithiasis | | + | 1.4% (2) | 23.8% (35) | 22.4% (33) | 81.6% (120) |
|  |  | - | 3.9% (8) | 28.9 %(60) | 25.1% (52) | 85.9% (178) |
|  |  | p | 0.28 | 0.34 | 0.65 | 0.34 |
| Antibiotic treatment within past 3 months | + | | 3.1% (5) | 30.5% (50) | 27.4% (45) | 85.4% (140) |
|  | - | | 2.6% (5) | 23.7% (45) | 21.1% (40) | 83.2% (158) |
|  | p | | 1 | 0.18 | 0.20 | 0.67 |
| Hospital stay within the past 6 months | Yes | | 3.2% (7) | 29.8% (65) | 25.7% (58) | 87.2% (190) |
|  | No | | 2.2% (3) | 22.1% (30) | 21.3% (27) | 79.4% (108) |
|  | p | | 0.12 | 0.14 | 0.42 | 0.07 |
| In-situ catheter at the time of diagnosis |  | |  | | | |
|  | Catheter in-situ at diagnosis of urosepsis for more than 48 hours | Yes | 5.4% (8) | 33.8% (50) | 28.4% (42) | 91.9% (136) |
|  |  | No | 0.9% (2) | 21.8% (45) | 20.9% (43) | 78.6% (162) |
|  |  | p | 0.03 | **0.01** | 0.13 | **0.00** |
|  | Urethral | + | 9.9% (7) | 40.8% (29) | 30.9% (22) | 92.9% (66) |
|  |  | - | 1.1% (3) | 23.3% (66) | 22.3% (63) | 81.9% (232) |
|  |  | p | 0.00 | **0.00** | 0.16 | **0.03** |
|  | Suprapubic | + | 0 (0) | 33.3% (3) | 33.3% (3) | 100% (9) |
|  |  | - | 2.9% (10) | 26.7% (92) | 23.8% (82) | 83.8% ( 289) |
|  |  | p | 1 | 0.94 | 0.78 | 0.39 |
|  | Nephrostomy | + | 3.3% (1) | 23.3% (7) | 20.0% (6) | 90% (27) |
|  |  | - | 2.9% (9) | 27.2% (88) | 24.4% (79) | 83.6% (271) |
|  |  | p | 1 | 0.81 | 0.75 | 0.51 |
|  | Ureteral stent | + | 2.2% (1) | 30.4% (14) | 28.3% (13) | 91.3% (42) |
|  |  | - | 2.9% (9) | 26.3% (81) | 23.4% (72) | 83.1% (256) |
|  |  | p | 1 | 0.68 | 0.59 | 0.23 |
| Previous urinary tract intervention | + | | 6.3% (9) | 32.9% (47) | 26.6% (38) | 84.6% (121) |
|  | - | | 0.5% (1) | 22.7% (48) | 22.2% (47) | 83.9% (177) |
|  | p | | **0.00** | **0.04** | 0.42 | 0.97 |
| Health care associated infection | + | | 1.5% (2) | 26.7% (35) | 25.2% (33) | 88.5% (181) |
|  | - | | 3.6% (8) | 26.9% (60) | 23.3% (52) | 81.6% (116) |
|  | p | | 0.42 | 1 | 0.78 | 0.11 |

~Imputation of missing variables did not make a difference to the findings.

Ϫ Imputation of missing variables made a difference to the findings and p value dropped below threshold of 0.05.

### Risk factors of outcomes adjusted for sepsis severity and their relationship with MDR bacteria in urine

###

s-Table 5. Resistance profile of urine bacteria in risk factors associated with unfavourable outcomes of urosepsis.

|  | | Pan susceptible | Resistant to ≥1 agent in <3 antimicrobial categories | Multi-drug resistant | p value |
| --- | --- | --- | --- | --- | --- |
| Age | | 62.1 (17.6) | 62.1 (17.6) | 61.7 (14.4) | >0.1 |
| Charlson comorbidity index score | | 1.3 (1.8) | 1.7(2.4) | 1.6 (1.9) | 0.00 |
| Urinary tract obstruction at the time of diagnosis | Yes | 40.3% | 33.7% | 25.9% | 0.46 |
|  | No | 39.1% | 28.9% | 32.0% |  |
| Urethral catheter is-situ | Yes | 28.8% | 30.3% | 40.9% | 0.01 |
|  | No | 42.8% | 32.1% | 25.1% |  |
| Previous urinary tract intervention | Yes | 28.3% | 34.2% | 37.5% | 0.00 |
|  | No | 47.1% | 30.2% | 22.8% |  |
| Health care associated infection | Yes | 30.4% | 34.8% | 34.8% | 0.03 |
|  | No | 45.2% | 29.9% | 24.9% |  |
| ICU | Yes | 25.6% | 25.6% | 48.8% | 0.00 |
|  | No | 42.1% | 32.7% | 25.2% |  |

# SERPENS Study Investigators

| **Site** | **Investigator/s** | **Country** | **Number of patients that entered final analysis** | **Attrition rate** |
| --- | --- | --- | --- | --- |
| **South Pest Teaching Hospital** | Bela Koves | Hungary | 44 | 53% |
|  | Peter Tenke |  |  |  |
| **Surgical Clinic St. Naum Ohridski, Skopje** | Slobdan Ristovski | Macedonia | 46 | 3% |
|  | Maja Sofronievska Glavinova |  |  |  |
| **Gaziosmanpasa Taksim Teaching Hospital, Istanbul** | Mustafa Bahadir Can Balci | Turkey | 28 | 33% |
|  | Baris Nuhoglu |  |  |  |
|  | Bugra Cetin |  |  |  |
| **Urology Department, Oslo University Hospital** | Kristin Rennesund | Norway | 28 | 24% |
|  | Truls Erik Bjerklund Johansen |  |  |  |
| **Univeristy Hospital of Larissa** | Stavros Gravas | Greece | 27 | 10% |
|  | Michael Samarinas |  |  |  |
| **Clinical University Center Sarajevo** | DjordJe Nale | Serbia | 24 | 20% |
|  | Branka Terzic |  |  |  |
| **Hospital Universitario 12 de Octubre** | Jose Medina Polo | Spain | 20 | 29% |
| **Teaching Hospital Trenčín** | Maria Garabasove | Slovakia | 20 | 16% |
|  | Maria Štefkovičová |  |  |  |
|  | Maria Kopilec Garabasov |  |  |  |
| **Ospedale Santa Maria della Misericordia di Perugia** | Elisabetta Costantini | Italy | 16 | 33% |
|  | Ester Illiano |  |  |  |
| **Hospital General Universitario  Gregorio Marañón, Madrid** | Jorge Cano-Valasco | Spain | 14 | 50% |
| **CHRU Tours** | Franck Bruyere | France | 11 | 21% |
| **S.R. Institute of Urology, Moscow** | Tamara Perepanova | Russia | 8 | 60% |
|  | Peter Hasan |  |  |  |
| **Novosibirsk Research Institute of Tuberculosis** | Ekaterina Kulchavenya | Russia | 4 | 0 |
| **Trakya Medical School, Edirne** | Mete Cek | Turkey | 2 | 75% |
| **Justus Liebig University, Giessen** | Florian Wagenlehner | Germany | 7 | 36% |
| **Centro Hospitalar Cova de Beira, Lisbon** | Bruno Pereira | Portugal | 3 | 88% |
| **National University Cancer Institute, Singapore (NCIS)** | Fiona Wu | Singapore | 2 | 50% |
| **Nicolaus Copernicus City Hospital Specjalistyczny Szpital Miejski, Torun** | Przemyslaw Adamczyk | Poland | 4 | 20% |
| **Marmara University Pendik Training and Research Hospital, Istanbul** | Yiloren Tanidir | Turkey | 2 | 67% |
| **University Clinical Center Tuzla** | Mustafa Bazardzanovic | Bosnia and Herzegovina | 11 | 21% |
| **Sana Klinikum Hof GmbH** | Hansjörg Keller | Germany | 5 | 16% |
|  | Razvan-Vasile Dican |  |  |  |
| **Hospital de Braga** | Carlos Oliviera | Portugal | 8 | 20% |
| **San Luca Nuovo Padiglione, Florence** | Andrea Cocci | Italy | 1 | 50% |
| **La Paz University Hospital, Madrid** | Juan Gomez Rivas | Spain | 5 | 72% |
| **Ospedale Ca’ Foncello di Treviso** | Francesco Beniamin | Italy | 2 | 33% |
| **EstiMed, Tashkent** | Seydali Eredjepov | Uzbekistan | 1 | 50% |
| **Ivanovo Regional Clinical Hospital** | Alexey A. Shevyrin | Russia | 2 | 100% |
| **Clinical University Center Sarajevo** | Senad Bajramovic | Bosnia and Herzegovina | 1 |  |
| **Regina Apostolorum Hospital S. Evyenio, Rome** | Tommaso Brancato | Italy | 2 | 80% |
| **General Hospital of Bolzano** | Christian Ladurner | Italy | 2 | 50% |
| **Hospital Valle del Nalón** | Miguel Alvarez Mugica | Spain | 3 |  |
| **Landesklinikum Baden** | Claus Riedl | Austria | 1 | 50% |

1. Wagenlehner F, Tandogdu Z, Bartoletti R, et al. The Global Prevalence of Infections in Urology Study: A Long-Term, Worldwide Surveillance Study on Urological Infections. Pathogens (Basel, Switzerland) 2016;5.
